# Compensatory increases in tear volume and mucin levels associated with meibomian gland dysfunction caused by stearoyl-CoA desaturase-1 deficiency

**DOI:** 10.1038/s41598-018-21542-3

**Published:** 2018-02-20

**Authors:** Takaaki Inaba, Yasuhisa Tanaka, Shusaku Tamaki, Tomotaka Ito, James M. Ntambi, Kazuo Tsubota

**Affiliations:** 1 Department of Ophthalmology, Keio University School of Medicine, 35 Shinanomachi, Shinjuku-ku, Tokyo 160-8582 Japan; 2 Departments of Biochemistry and of Nutritional Sciences, University of Wisconsin-Madison, Madison, WI 53706 USA

## Abstract

The stearoyl-CoA desaturase (SCD) family of enzymes catalyzes monounsaturated fatty acid synthesis by inserting a *cis* double bond at the Δ9 position of saturated fatty acids. Disruption of these enzymes has been reported to induce a severe dry skin phenotype. Since lipid abnormalities in the meibomian glands have been associated with dry eye, we analyzed selected eye tissues contributing to tear volume and composition in genetically SCD-1-deficient mice (SCD-1 KO), including the lacrimal glands and conjunctiva. Previous histopathological analysis had revealed atrophy and loss of meibomian glands; taken together with the increased goblet cell and MUC5AC expression in the conjunctiva reported here, these findings suggest that the tear volume and mucin levels secreted are enhanced in the absence of lipid secretion as a compensatory mechanism. The expression of lipid metabolism genes in lacrimal glands was decreased in SCD1 KO mice. Thus, these results provide new pathophysiological mechanisms to pursue with regard to meibomian gland dysfunction. In addition, lack of SCD-1 causes a compensatory increase in the tear volume and mucin levels associated with changes in expression of lipid metabolism genes. These results may be useful as a new concept for dry eye treatment strategies.

## Introduction

Meibomian gland dysfunction (MGD) is a chronic or pervasive abnormality of the meibomian glands, in which qualitative and quantitative changes to glandular secretions occur in association with obstruction of the gland ducts^1–4^. Further, these obstructions may cause changes in tear layer function, ocular discomfort, severe inflammation of the ocular surface, or epithelial disorders^5^. The condition presents with diverse clinical profiles that may or may not involve inflammation, resident bacteria, and show different degrees of disease severity, ranging from mild to severe. However, at present, there are few effective treatments for MGD.

Previous reports have indicated that stearoyl coenzyme A desaturase-1 deficient (SCD-1 KO) mice exhibit meibomian gland abnormalities^6^. The enzyme SCD-1 plays a central role in lipid synthesis in the sebaceous and meibomian glands by introducing a double bond into the saturated fatty acids stearic acid and palmitic acid, thereby inducing the production of oleic acid (18:1) and palmitoleic acid^7–9^. SCD-1 KO mice have also been reported to exhibit dyslipidemia-induced dry skin and atrophy of the meibomian glands^6,10–14^. The tear film consists of three layers. The lipid, aqueous, and mucin layers of the tear film affect each layer of the ocular surface. We hypothesized that if any one of the three layers was lacking, this may result in a compensatory response. Therefore, in this study, we aimed to ascertain the effect of meibomian gland abnormality on dry eye by analyzing tears and ocular tissue in SCD-1 KO mice.

## Results

### SCD-1 expression in the lacrimal glands

**Figure 1:**
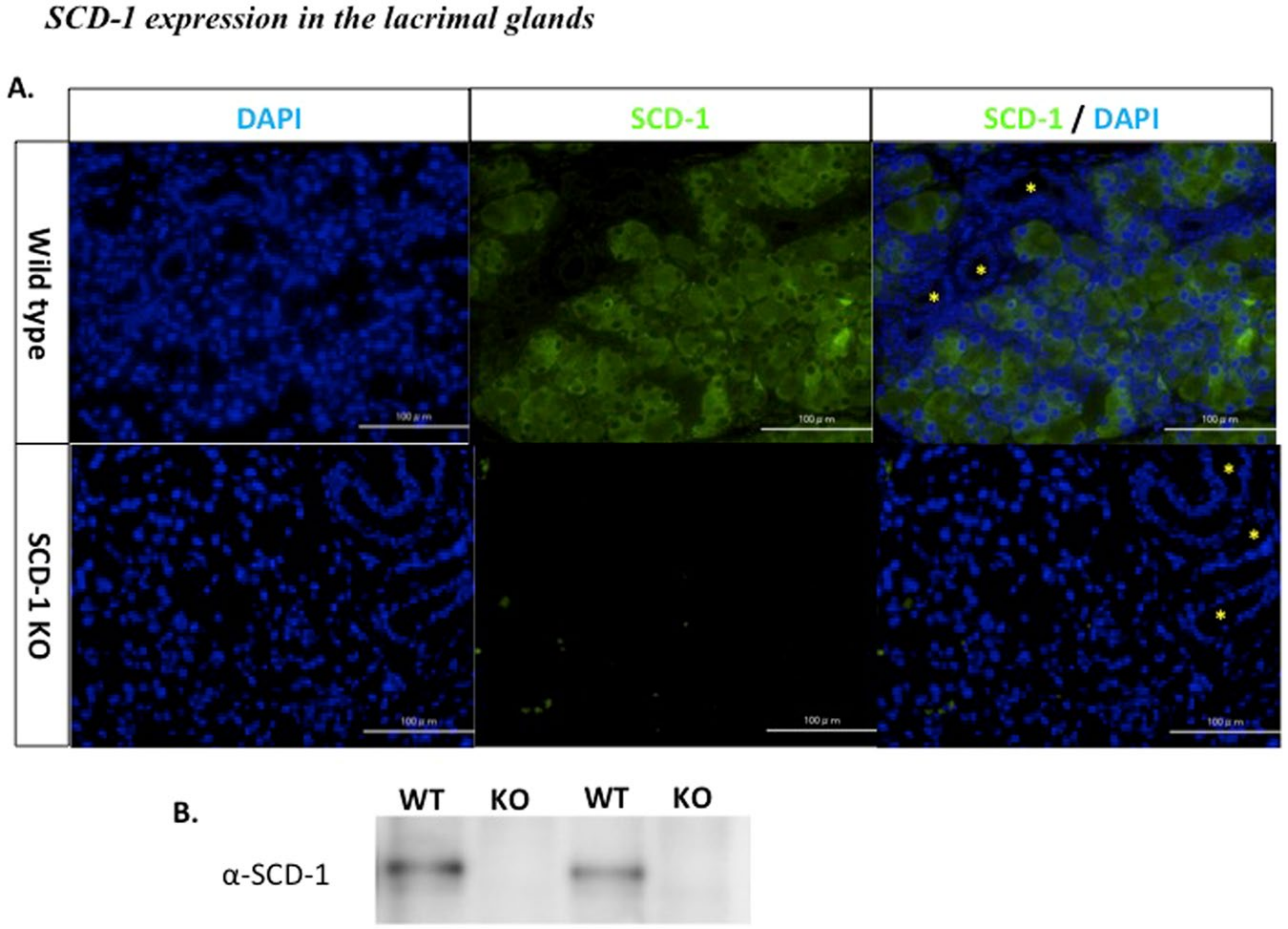
SCD-1 expression in the lacrimal glands. (**A**) Immunohistochemical localization of SCD-1 in the lacrimal glands of wild-type and SCD-1 KO mice. The panels indicate DAPI (blue), SCD-1 (green), and the merged image(*; ductal cells, scale bar: 100 µm). (**B**) Western blot analysis of lacrimal glands from wild-type and SCD-1 KO mice using anti- SCD-1 antibodies. Samples derive from the same experiment and gels/blots were processed in the same gel. All experiments were performed at least three times with similar results, and representative data are shown.

The SCD-1 expression in lacrimal glands and its contribution to tear secretion have not previously been elucidated. SCD-1 localization in the lacrimal glands was evaluated by immunohistochemical analysis and western blotting. The immunohistochemical results indicated that SCD-1 was localized in the cytoplasm of acinar cells in wild-type mice, but was not detectable in ductal cell of wild type and LG of SCD-1 KO mouse (Fig. 1A). Western blot analysis revealed that SCD-1 expression was absent in SCD-1 KO mice in contrast to LG from wt mice, which expressed an immunopositive band (Fig. 1B).

### Changes in the tear volume of SCD-1 KO mice

**Figure 2:**
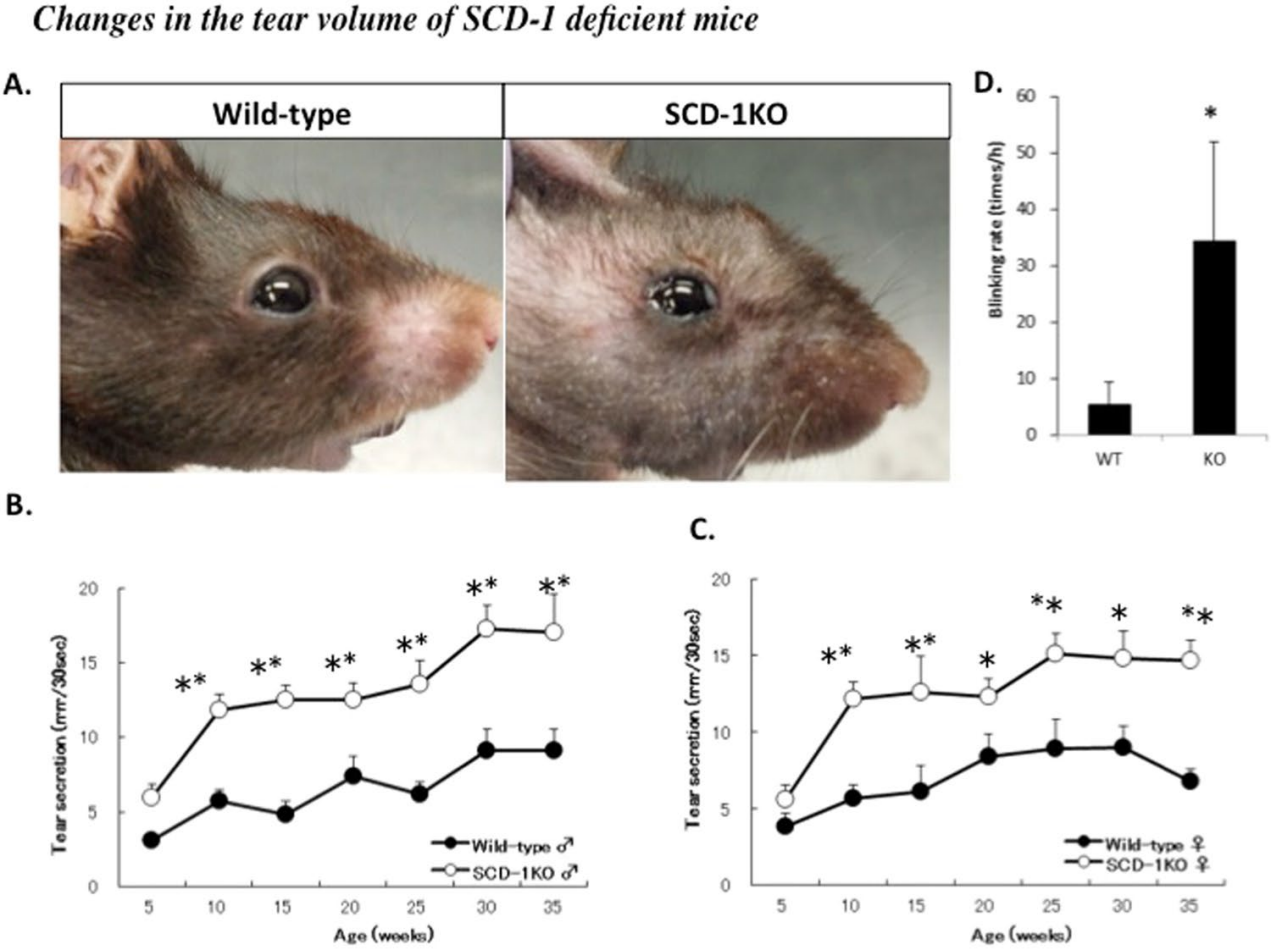
Changes in the tear volume of SCD-1 KO mice. (**A**) Images of the anterior ocular surface. In the SCD-1 KO mice, abundant tears were viewed and photographed under white light. (**B**,**C**) Basal tear volume of wild-type (n = 8) and the SCD-1 KO mice (n = 8) at 5 weeks and 35 weeks. Male: B, Female: C. (**D**) Comparison of blinking rate was counted under normal conditions at 10 weeks. All experiments were presented as means ± SEM. Student’s t-test, **P < 0.01; *P < 0.05 versus wild-type.

It was evident by visual inspection that the ocular surfaces of the SCD-1 KO mice were lubricated with more abundant tears compared to those of wild-type mice (Fig. 2A). The tear volume of the wild-type and the SCD-1 KO mice was assessed using the cotton thread test. The results showed a significantly higher basal tear volume in SCD-1 KO mice compared to that in wild-type mice at 5–35 weeks of age (Fig. 2B). The increase in tear volume was observed in both male and female mice (Fig. 2C). Moreover, SCD-1KO mice showed increased blinking rates because of the ocular surface instability with a lipid layer defect (Fig. 2D).

### Changes in the conjunctiva of SCD-1 KO mice

**Figure 3:**
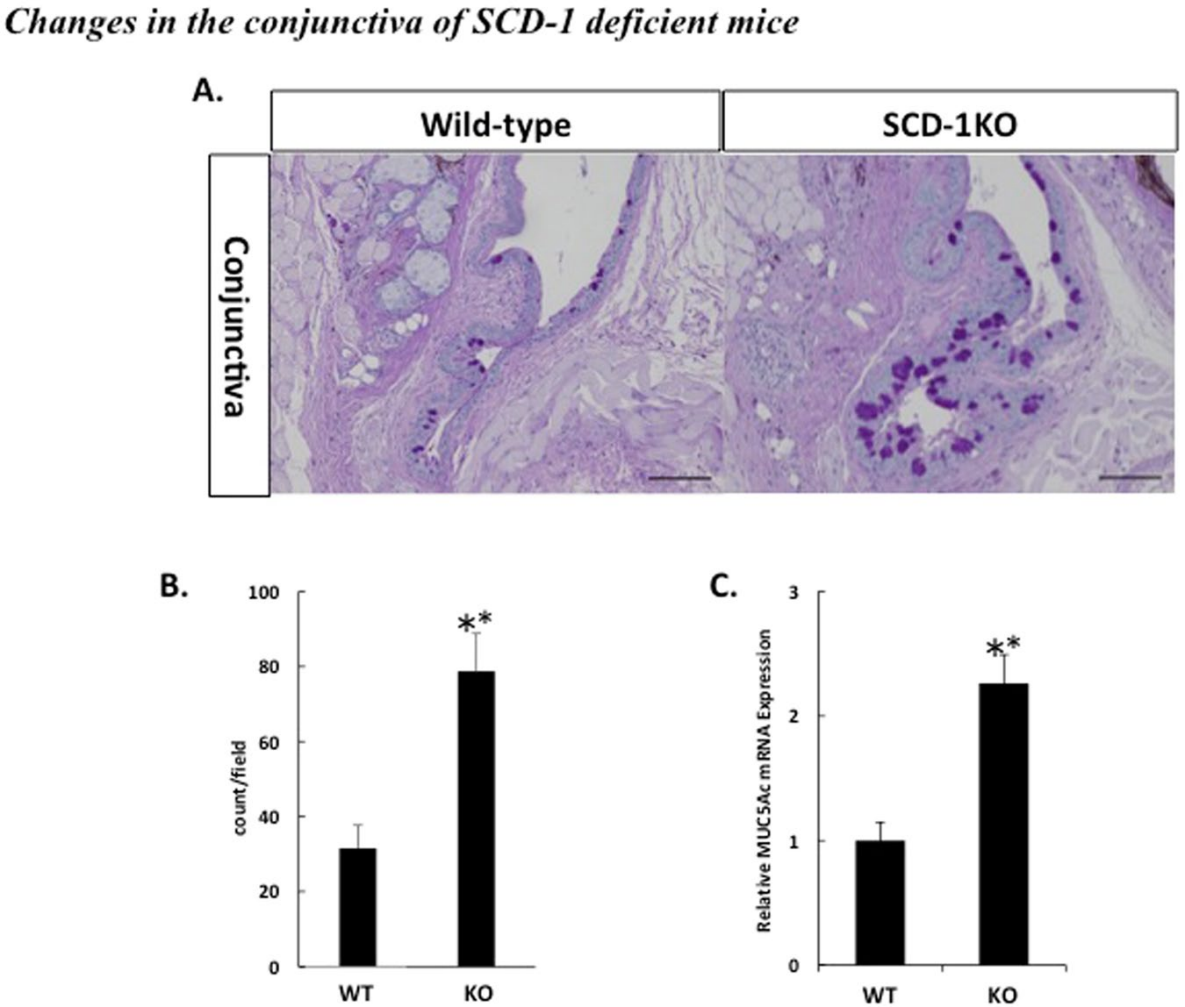
Changes in the conjunctiva of SCD-1 KO mice. (**A**) Tissue sections of conjunctiva from wild-type and SCD-1 KO mice were stained by PAS and observed by light microscopy using bright field. Scale bar: 100 µm. (**B**) Relative goblet cell number. The goblet cells in the conjunctiva of wild-type (n = 8) and SCD-1 KO mice (n = 10) were measured using PAS-stained sections. (**C**) Real-time PCR analysis of conjunctiva from wild-type and SCD-1 KO mice. All data are presented as means ± SEM. Student’s t-test, **P < 0.01 versus wild-type. All experiments were performed at least three times with similar results, and representative data are shown.

Conjunctival tissue sections were stained with Periodic Acid Schiff (PAS) (Fig. 3A) and the goblet cells were counted. The results revealed a significant increase in goblet cells in SCD-1 KO mice compared with the cell numbers in wild-type mice (Fig. 3B). To further investigate this increase, the MUC5AC mRNA levels of the conjunctiva of wild-type and the SCD-1 KO mice were measured using qRT-PCR. The results indicated significantly higher abundance, normalized to beta actin, of MUC5AC mRNA in the conjunctiva of SCD-1 KO mice (Fig. 3C), which was consistent with the greater number of goblet cells. We also observed MGD in SCD-1 KO mice, which were consistent with the findings in previous reports^6^ (Results not shown).

### Changes in the lacrimal glands of SCD-1 KO mice

**Figure 4:**
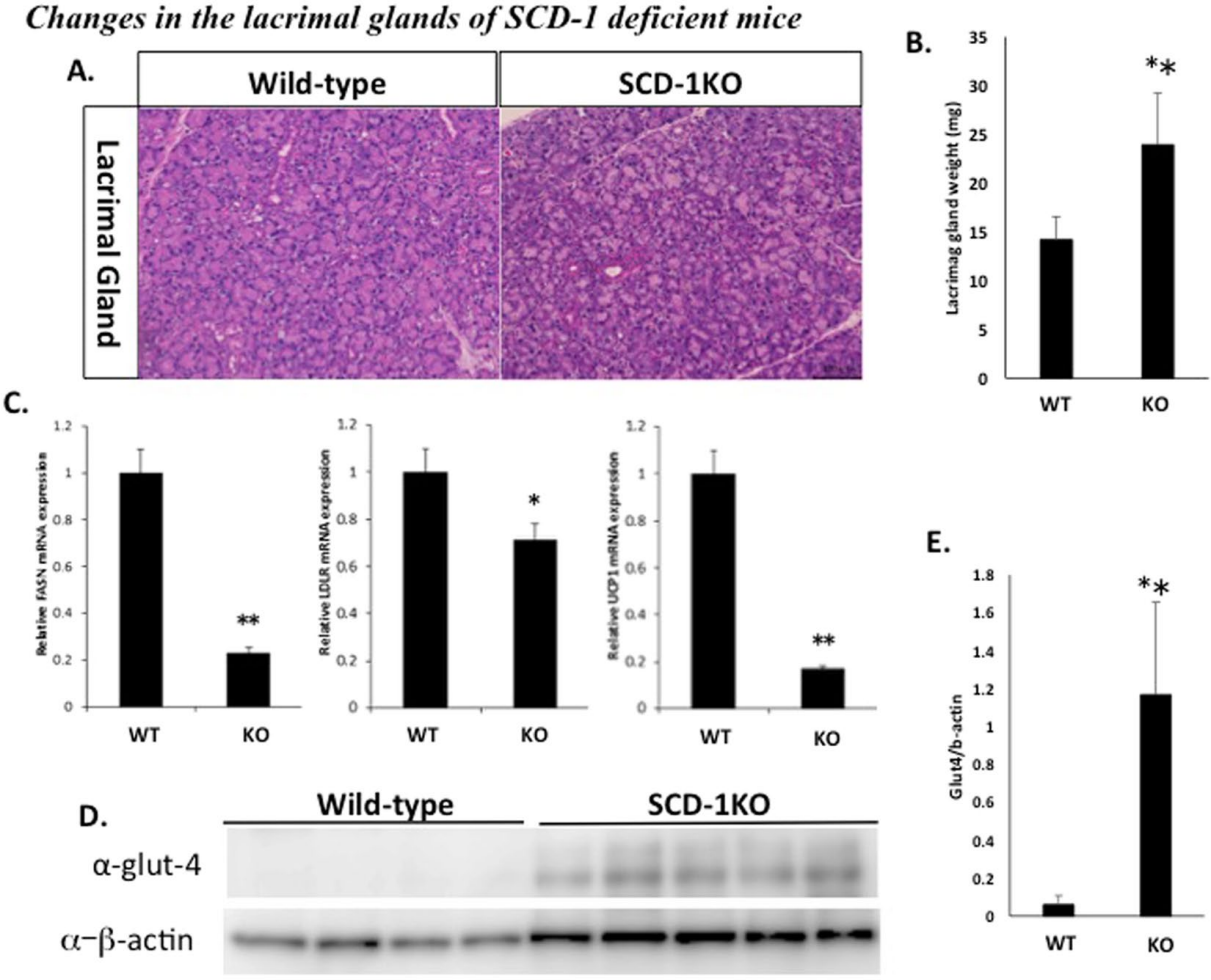
Changes in the lacrimal glands of SCD-1 KO mice. (**A**) Tissue sections of lacrimal glands from wild-type and SCD-1 KO mice were stained by hematoxylin/eosin (HE) and observed under light microscopy. (**B**) Average lacrimal gland weights of wild-type and the SCD-1 KO mice. (**C**) Real-time PCR analysis of lacrimal glands from wild-type and SCD-1 KO mice. The SCD-1 KO mice displayed decreased FASN, LDLR and UCP-1 expression compared to wild-type. (**D**) Western blot analysis of lacrimal glands from wild-type and SCD-1 KO mice, using Glut4 and beta-actin antibodies. Samples derive from the same experiment and both samples (WT and SCD-1 KO) were processed in the same gel. β-Actin was used as the control (cropped band shown) and gels/blots were processed in parallel. (**E**) Relative protein levels of Glut4 versus beta-actin in whole cell lysate. All data are presented as means ± SEM. Student’s t-test, **P < 0.01; *P < 0.05 versus wild-type. All experiments were performed at least three times with similar results, and representative data are shown.

A possible mechanism for the increased tear volume in the lacrimal gland was investigated by analyzing changes in the LG as part of a detailed assessment of SCD-1 KO mice. H/E staining of LG sections in wild-type and SCD-1 KO mice did not show any differences (Fig. 4A). However, the freshly dissected wet weight of the lacrimal glands was significantly greater in SCD-1 KO mice when compared to that in wild-type mice (Fig. 4A and B). This increase supports the observed tear secretion phenotype in SCD-1 KO mice (Fig. 2A–C)^15^. In SCD-1 KO mice, the expression levels of lipid metabolism genes such as FASN, LDLR and UCP-1 were determined by qRT-PCR analysis. Expression of all of these genes was decreased in statistically significant fashion vs. WT values in the lacrimal gland of SCD-1 KO mice (Fig. 4C). Furthermore, it was found using WB analysis that glut4 protein expression in the lacrimal gland was significantly higher in SCD-1 KO mice than in WT mice (Fig. 4D and E).

## Discussion

**Figure 5:**
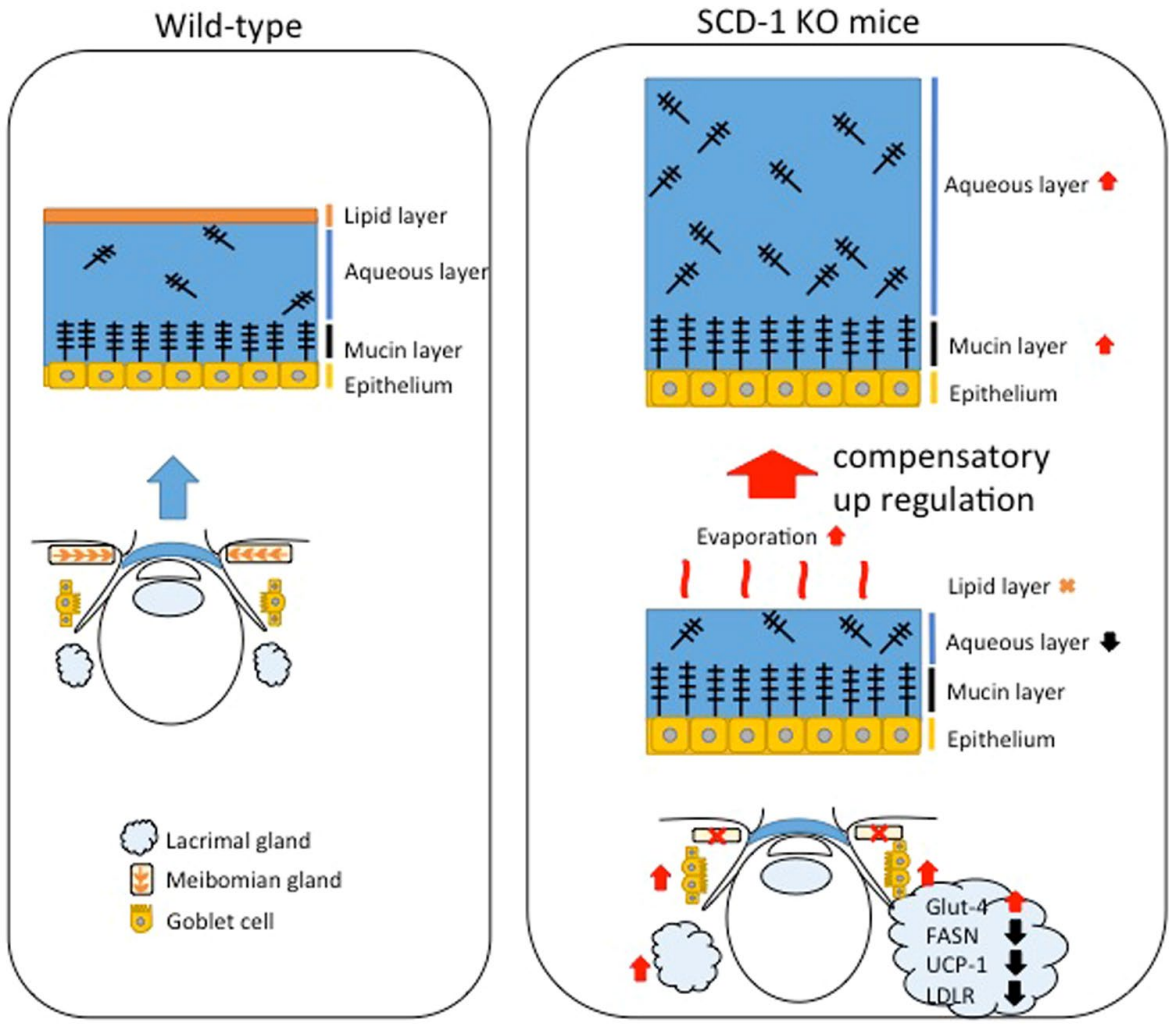
Schema of compensation mechanism. The normal tear film consists of three layers that maintain the tear film stability (Wild-type). SCD-1 KO mice exhibit meibomian gland dysfunction. Since the lipid layer was decreased, water evaporates gradually and the aqueous layer becomes thin. The SCD-1 KO mice showed an accelerated compensation mechanism to protect the corneal epithelium. As a result, increased secretion of tear fluid and mucins were associated with elevated expression of lipid metabolism genes such as FASN, LDLR, and UCP-1 in SCD-1 deficient mice.

Through production and secretion of lipids, meibomian glands promote the stability and prevent the evaporation of the tear film. We examined the tear film, as well as two tissue sources of tear film (the lacrimal gland and conjunctival goblet cells) in SCD-1 KO mice. Our results showed that lack of SCD-1 results in an increase in the tear volume and cellular mucin levels, and raises the question of whether these changes are associated with deranged lipid metabolism, as are demonstrated here in SCD-1 KO mice (Fig. 5). SCD-1 was localized to the cytoplasm of acinar cells of the LG, and SCD-1 KO mice showed a higher basal tear volume, a significant increase in the number of goblet cells, and an increase in the weight of the lacrimal glands.

The increase of tears observed in SCD-1 KO mice suggests that lipids may be involved in the regulation of the aqueous component of tear secretion by the LG. Previous studies have shown that the amount of tears is lower in obese or diabetic mice, which indirectly suggests that excessive lipid levels reduce tear volume^16,17^. Other reports have indicated that SCD-1 KO mice are resistant to obesity and exhibit little fatty tissue accumulation^18,19^. These findings imply that lowered levels of lipid metabolism may either compensatory increase the amount of tears by a currently unknown mechanism, or reduce the expression of tear-suppression factors^20^. There is some connection between tear production and metabolic syndrome in humans^21^. Lack of SCD-1 decreased expression of other lipid metabolism genes in the lacrimal gland. Furthermore, expression of transporter-associated proteins such as glut-4 was also elevated, which could be the mechanism underlying the apparent compensatory increased tear volume. Glut-4 has several features common to the regulated exocytosis pathway of secretory vesicles, including the v-SNARE (soluble N-ethylmaleimide-sensitive factor attachment protein receptor), VAMP2, and the t-SNARE, syntaxin-4. Tear proteins are released by means of the regulated fusion of secretory vesicles at the apical surface of lacrimal gland acinar cells. Since secretory vesicles of lacrimal glands contain nutrients and protein (such as lacritin and EGF), antibacterial and antiviral factors (such as IgA and lactoferrin), and glucose, tear secretion may be related to this effect^22–24^.

Tear secretion is regulated by the lacrimal functional unit, which consists of the cornea, conjunctiva, lacrimal glands, meibomian glands, and the nerves (parasympathetic, sympathetic and sensory)^25–27^. The condition of this functional unit plays an important role in maintaining the quality of vision. Slight changes in the tear film, such as reduced lacrimal gland secretion, can lead to dry eye disease^28^. The tear film consists of three layers. The lipid, aqueous, and mucin layers of the tear film affect each layer of the ocular surface. We hypothesized that if any one of three layers was lacked, a body does compensate for the lack of this. The dry eye society recently initiated the concept of Tear Film Oriented Therapy (TFOT). The purpose of TFOT is to target layers on the ocular surface via topical therapy^29–31^.The optimal treatment approach for an abnormal layer is to improve the integrity and function of the other two layers. TFOT initiates a positive cycle involving the tear film and superficial epithelium, and the lacrimal functional units, meibomian gland, and conjunctiva contribute to this cycle. Our result demonstrated that this positive cycle may encompass increased mucin expression and tear volume in SCD-1 deficiency with MGD.

Catalytic saturation of tear film lipid layer (TFLL) is demonstrated *in vitro* to alter the thermotropic behavior and to inhibit the uniform spreading of TFLL^32,33^ which in turn is shown clinically to be a major feature for a normal tear film^34^. It is also hypothesized that MUC5AC and mucomimetic polymers can act as a spreading agent for TFLL^35^. Thus increase of MUC5AC content can partially compensate for impaired spreading due to partial loss of lipid unsaturation. The possible interplay between the compounds is also indicated by data that in the tear film of neonates there is excess of lipids and of MUC5AC that contributes to the much higher TF stability^36^. The same is valid for the increase of aqueous tear volume that is well known to facilitate the lipid spreading^34,37^. Furthermore MUC5AC can increase the viscosity of the tear at low shear rates (during aqueous tear establishment after eye opening and at partial blink) which is known to increase the TF deposition thickness and breakup time^38^. Also MUC5AC can enhance the structure of the mucoaqueous layer and increase its mechanical stability^39,40^. Thus the increase of MUC5AC level and of aqueous tear volume look to precisely compensate for the possible impaired spreading of TFLL due to lowered degree of acyl chain unsaturation. Such single paragraph discussion is useful to guide the reader among the current state of the art in the field and also allows perceiving the modern understanding that the layers of the tear film act in mutual relationship and not as isolated entities.

The lipid-secretory organs, such as the meibomian gland in humans and the harderian gland in mice were anatomically different between humans and mice. The results of the current study indicated that SCD-1 KO mice developed MGD^6^. Patients with dry eye caused by MGD may often exhibit tear production that is within normal ranges of secretion rate, composition and tear volume^41^. In such patients the MGD may elicit a compensatory mechanism affecting the LG and goblet cells that prevents the tear volume from declining. Considering this hypothesis and the reports of decreased lipid synthesis in the meibomian glands of SCD-1 KO mice^6^, it is possible to suggest that the SCD-1 KO mice model develops MGD-like symptoms, thereby stimulating a mechanism that protects against the decline in meibomian gland secretion through excessive tear production. Indeed, such an increased compensatory mechanism of tear increase has been observed in human dry eye patients with MGD^31^.

In conclusion, our results suggest that lack of SCD-1 causes a compensatory increase in the tear volume and mucin levels through its effects on lipid metabolism as detailed here in SCD-1 KO mice. Further studies are necessary to establish further mechanistic details of a proposed compensatory mechanism.

## Methods

### Experimental Animals

All the animal experiments were conducted in accordance with the Association for Research in Vision and Ophthalmology (ARVO) Statement for the use of animals in research. All experiments were approved by the Japanese Physiological Society’s guidelines for animal care and the animal experimental committees at KEIO University. Mice were maintained on a 12-hr light–dark cycle, with the dark cycle occurring from 8:00 P.M. to 8:00 A.M in a specific-pathogen-free environment. SCD-1 deficient (SCD-1 KO) mice^6^ with a C57BL/6 J background (B6.129-Scd1tm1Ntam/J;006201) were purchased from Jackson Laboratory (Bar Harbor, ME, USA). Tears and tissues were collected from all mice upon sacrifice at between 5 and 40 wk of age. The freshly dissected lacrimal gland (LG) wet weights were then measured, and the mean values were calculated to obtain the average LG weight of the mice.

### Measurement of tear volume

Basal tear secretion volume was measured with a cotton thread test by using a phenol red-impregnated thread (Zone-Quick, Showa Yakuhin Kako, Tokyo, Japan). The mice were restrained without anesthesia, and a cotton thread was placed on the temporal side of the lower eyelid margin for 30 s. The length of the moistened fragment was measured. Measurements were obtained for both the right and the left eyes.

### Immunohistochemical analysis

SCD-1 expression in LG histological sections from both wild-type and SCD-1 KO mice was detected immunohistochemically. The isolated LG were embedded in optimal cutting temperature (OCT) compound (Sakura Finetechnical, Tokyo, Japan) and fresh-frozen on ice. 5-μm thick frozen sections on glass slides were fixed with 10% neutral buffered formalin (NBF) (Wako, Osaka, Japan). Rinse briefly with PBS. Permeabilize with 0.5% Triton X-100 for 10 minutes. The glass slide**s** were blocked with 10% bovine serum albumin (BSA) and incubated with mouse monoclonal primary antibody against SCD-1 (1:1000; Cell Signaling Technology, Beverly MA, USA) overnight at 4 °C. Following washes with Tris-Buffered Saline + Tween-20 **(**TBST**)**, sections were incubated with rabbit anti-mouse IgG antibody conjugated to Alexa 555 (Dako, Glostrup, Denmark). Nuclear staining was performed with mounting medium containing 4′,6-diamidino-2-phenylindole (DAPI; Dojindo, Kumamoto, Japan). Immunofluorescent signals were detected by viewing samples in a fluorescent microscope (BZ-H1M analyzing system; Keyence, Osaka, Japan).

### Histopathology

For histopathological analysis, the isolated lacrimal glands were embedded in OCT and sectioned as described above. Frozen sections were fixed with NBF and stained with hematoxylin and eosin (H/E) or with the periodic acid-Schiff reagent.

### Measurement of goblet cell numbers in the conjunctiva

For quantitative analysis, 8 periodic acid-Schiff (PAS)-stained sections of the conjunctiva from wild-type and SCD-1 KO mice were used. The goblet cell number was measured using a previously reported approach^42^, by manually counting within 445 µm × 352 µm frames. Scores from the samples were averaged as the goblet cell density (number/µm^2^) for each group.

### Western blotting

LG were homogenized in Pro-Prep solution (iNtRON Biotechnology, Kyungki-Do, Korea), using mechanical dispersion (T-25; IKA, Stauffen, Germany). The protein concentration of the LG homogenate was measured using the BCA kit (Thermo Fisher Scientific, MA, USA) according to the manufacturer’s instructions. LG lysate was combined with sample buffer (125 mM Tris-HCl, pH 6.8; 20% glycerol; 4.0% sodium dodecyl sulfate [SDS]; 10% 2-mercaptoethanol; 0.1% bromophenol blue), and 1 µg of protein was separated by 12.5% SDS-polyacrylamide gel electrophoresis (PAGE) and transferred to a polyvinylidene difluoride membrane. The membrane was blocked with PVDF Blocking Reagent for Can Get Signal ® (Toyobo, Osaka, Japan) for 2 h and probed with the indicated primary antibodies. The primary antibodies SCD-1 (1:1000, Rabbit; Cell Signaling Technology, Beverly MA, USA), glut4 (1:1000, Rabbit; Cell Signaling Technology), and beta-actin (1:1000, mouse; Sigma, St. Louis, MO, USA) were used. Incubation of the membrane with primary antibody was performed overnight at 4 °C. After washing with (TBST), the membranes were further incubated with horseradish peroxidase-labeled secondary antibodies (1:1000; GE Healthcare, Amersham, UK) for 1 h at room temperature. The immobilized specific antigen was visualized with the ECL prime detection kit (GE Healthcare). Signal intensities were quantified using the ImageJ program and normalized to that of beta-actin.

### Quantitative Real-time PCR

Total RNA was extracted from the lacrimal gland or conjunctiva of mice by using the TRIzol reagent (Invitrogen, Carlsbad, CA, USA) according to the manufacturer’s instructions. Complementary DNA was produced from total RNA using RevertraAce Master Mix (Toyobo, Osaka, Japan). Quantitative real-time (qRT-)PCR was performed using the StepOne-Plus Real Time PCR system (Applied Biosystems, Foster City, CA, USA) with Fast Advanced Master Mix (Applied Biosystems) and using the predesigned primers for mucin5AC (MUC5AC), fatty acid synthase (FASN), low density lipoprotein receptor (LDLR), uncoupling protein-1 (UCP-1) and beta-actin [TaqMan Gene Expression Assay (MUC5AC: Mm01276718-m1,FASN: Mm00662319_m1, LDLR: Mm01177349_m1,UCP-1: Mm01244861_m1 and beta-actin: Mm00607939-s1)]. The mRNA levels were evaluated by the ΔΔCT method, and normalized to beta-actin mRNA.

### Statistical analysis

All summarized data were expressed as mean ± SEM. Statistical significance was calculated using the unpaired Student’s *t*-test. P values of less than 0.05% were considered statistically significant.

### Data Availability

All data generated or analyzed during this study are included in this published article.
